# Isolated Abducens Nerve Palsy Associated With Chikungunya: A Report of a Rare Case

**DOI:** 10.7759/cureus.71666

**Published:** 2024-10-16

**Authors:** Iqra Mushtaq, Kalibo Jakhalu, Tushar Agrawal, Lokisha Chandwani, Harshita Kashyap

**Affiliations:** 1 Ophthalmology, Dr. D. Y. Patil Medical College, Hospital and Research Centre, Dr. D. Y. Patil Vidyapeeth (Deemed to Be University), Pune, IND

**Keywords:** chikungunya, diplopia, mosquito borne infection, ophthalmology, sixth nerve palsy

## Abstract

Chikungunya is an arboviral disease transmitted to humans primarily through the bite of infected *Aedes* mosquitoes, characteristically causing high-grade fever with painful arthralgia. Ocular manifestations secondary to chikungunya infection are not very common but have been reported, including cranial nerve involvement. We report a case of a 60-year-old Indian female with left abducens nerve palsy secondary to chikungunya infection for which a complete ophthalmic evaluation was done. The patient was managed with intravenous steroids for five days and then shifted to oral steroids after which she significantly improved within two weeks of treatment.

## Introduction

Chikungunya is an acute viral disease characterized mainly by fever and painful arthralgia. It is caused by the chikungunya virus (CHIKV), a member of the *Alphavirus* genus, and is transmitted by mosquito vectors, primarily *Aedes aegypti* and *A. albopictus*, from the *Togaviridae* family.

According to the European Centre for Disease Prevention and Control, as of July 31, 2024, there have been 450,000 reported CHIKV cases and over 160 deaths worldwide, with India accounting for 69,395 of these cases [1]. The disease is primarily transmitted through bites from infected mosquitoes but can also spread through maternal-fetal transmission and blood contact. While the majority of cases resolve without significant sequelae, a range of neurological and ophthalmic complications can arise. Among these, cranial nerve palsies are relatively rare but noteworthy for their potential impact on the patient's quality of life [2,3].

Sixth nerve palsy, which results in weakness of lateral rectus eye movement and diplopia, is an unusual but documented manifestation of viral infections. The pathophysiology behind this complication in chikungunya is not fully understood but may involve a direct invasion of the nervous system or an immune-mediated response. Sixth nerve palsy can also occur due to trauma, ischemia, neoplasm, leukemia, migraine, pseudotumor cerebri, multiple sclerosis, and other conditions [4].

## Case presentation

**Figure 1:**
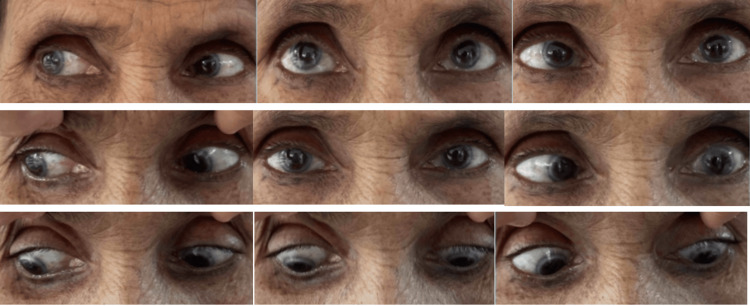
Clinical manifestations of left lateral rectus palsy through nine distinct gaze positions. Ocular motility examination revealed restricted movements in the left eye.

A 60-year-old lady presented to the outpatient department with complaints of insidious-onset fever, joint pain, and rashes over the past two weeks, followed by headache, diminished vision, and diplopia for the last five days. The best corrected visual acuity for distance was 20/60 in the right eye and 20/30 in the left eye. Ocular motility examination revealed restricted levo-elevation, abduction, and levo-depression in the left eye (Figure 1).

**Figure 2:**
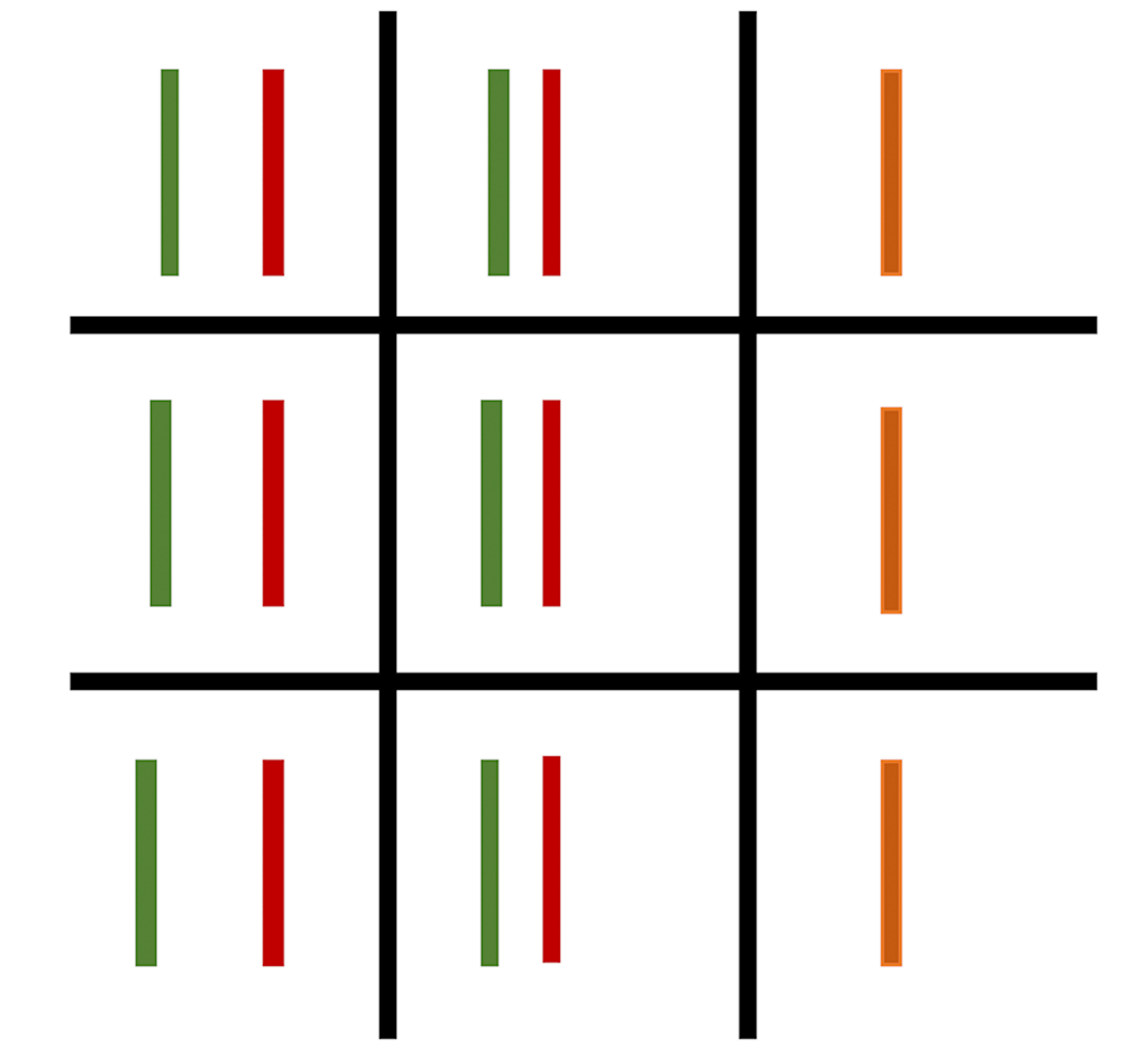
Diplopia charting Diplopia charting depicting left lateral rectus palsy, which gets worse on the left gaze. The red color indicates the image seen by the right eye, the green color indicates the image seen by the left eye and the orange color indicates the overlapping of the image seen by both eyes suggesting no misalignment.

Anterior segment examination performed using a slit lamp showed Grade 1 nuclear sclerosis. Pupils were equal, circular, and reactive to light, with both direct and consensual responses intact. A posterior segment examination conducted with an indirect ophthalmoscope and a 20D lens revealed mild temporal pallor. Visual field analysis was conducted using a Humphrey visual field analyzer (Carl Zeiss Meditec, Jena, Germany) and showed no abnormalities. Diplopia charting indicated left lateral rectus palsy (Figure 2).

**Figure 3:**
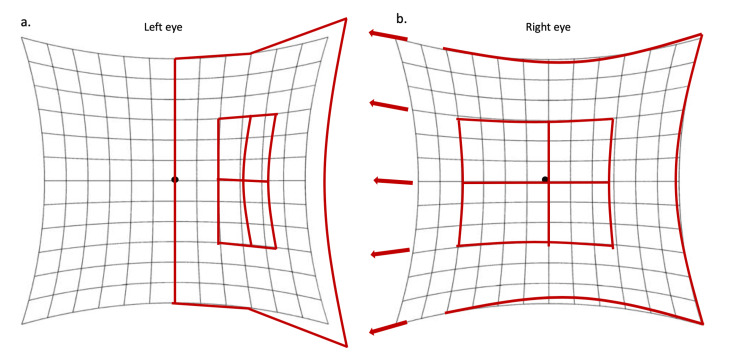
Hess charting of both eyes indicating left lateral palsy. Hess charting is done using red-green goggles with red on the right eye and green on the left eye. (a) Hess charting showing a smaller field on the left side when the left eye is abducting indicating under the action of the left lateral rectus. (b) Hess charting showing a larger field on the nasal side when the right eye is looking in the same direction, indicating over the action of the right medial rectus, due to Hering's law of equal innervation.

Hess charting indicated under the action of the left lateral rectus and over the action of the right medial rectus (Figure 3).

**Figure 4:**
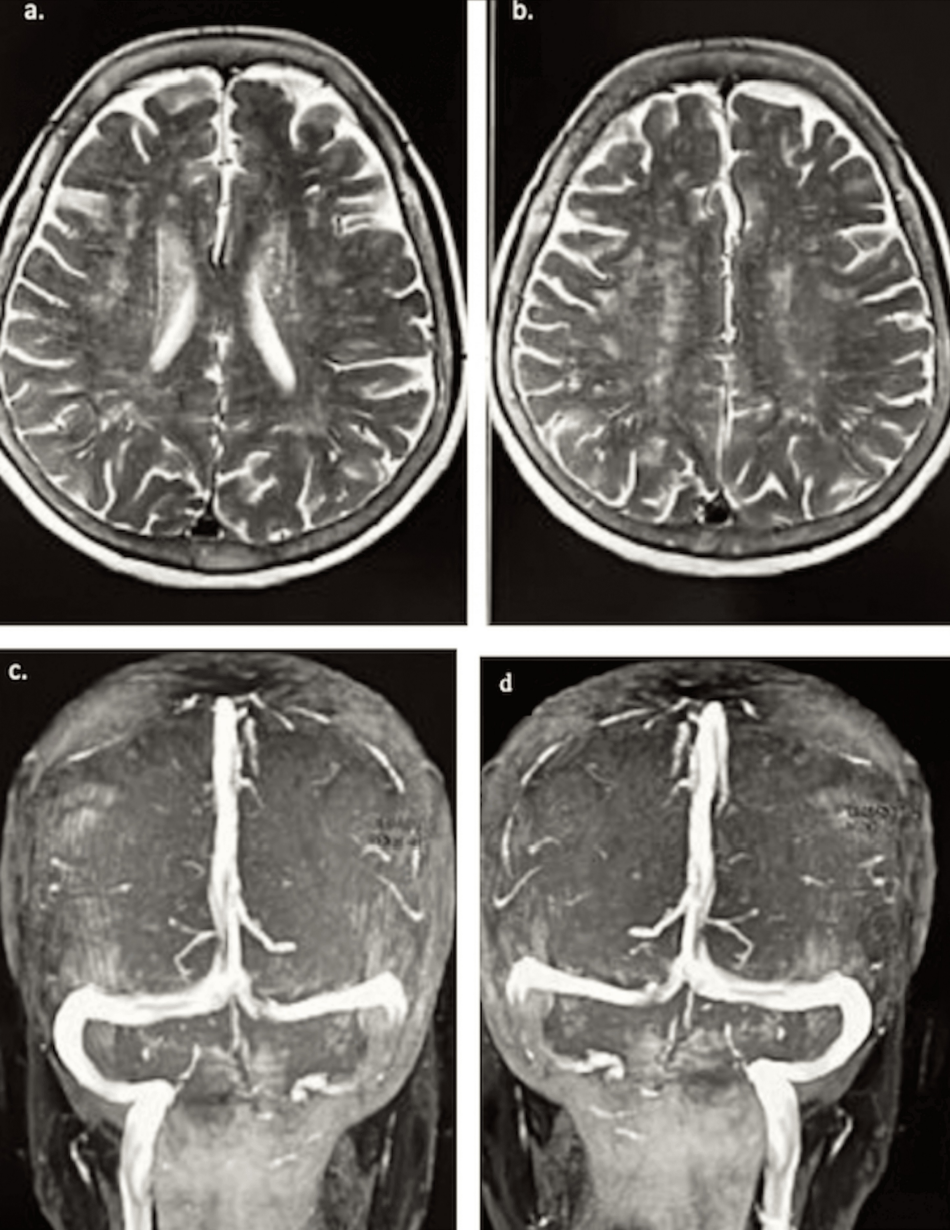
Magnetic resonance imaging (MRI) and magnetic resonance venogram (MRV) of the brain. (a, b) Axial plane section of MRI of the brain showing no abnormalities. (c, d) Coronal plane section of MRV of the brain showing no abnormalities.

A magnetic resonance imaging (MRI) scan and magnetic resonance venogram (MRV) of the brain were performed and were normal (Figure 4).

Upon systemic evaluation, the patient’s blood pressure was found to be 150/90 mmHg, for which antihypertensive medications were started. Routine investigations and a lipid profile were conducted, all of which were within normal limits. The erythrocyte sedimentation rate (ESR) and C-reactive protein (CRP) were also within normal ranges. A lumbar puncture was performed, and cerebrospinal fluid (CSF) analysis revealed clear and colorless fluid with normal opening pressure. The cell count was within normal limits, protein levels were slightly elevated (70 mg/dL), and glucose levels remained within the normal range. The absence of bacteria and the presence of elevated protein suggest an inflammatory response potentially attributable to chikungunya infection. Dengue and chikungunya serologies were conducted, with CHIKV serology testing positive for IgM by chromatographic immunoassay. The patient was planned for an injection of methylprednisolone 1 g for five days, followed by a shift to oral steroids at 40 mg/kg with tapering over one week. Significant clinical improvement was noted after two weeks of starting steroids.

## Discussion

CHIKV was first identified in 1952 in Makonde, a province in southern Tanzania, and has since spread to various regions, causing significant public health concerns [5]. CHIKV is classified as an "arthritogenic virus" because it causes inflammatory musculoskeletal diseases affecting multiple cell types, including dendritic cells, macrophages, synovial fibroblasts, endothelial cells, myocytes, and osteoblasts [6].

The symptoms of chikungunya typically begin within three to 12 days of being bitten by an infected mosquito. The infection is characterized by fever, generalized maculopapular rashes, myalgia, and polyarthralgia. It has three phases: acute, post-acute, and chronic. The acute phase lasts for the first three weeks and is characterized by fever, polyarthralgia/polyarthritis, intense myalgia, headache, photophobia, and rash. The post-acute phase begins in the third week and can last up to three months, featuring mild improvements in clinical condition and possible relapses. The chronic phase is defined by persistent arthralgia lasting beyond three months [7].

Ocular involvement secondary to chikungunya is uncommon. Some ocular manifestations include non-granulomatous and granulomatous anterior uveitis, panuveitis, optic neuritis, sixth nerve palsy with lagophthalmos, retrobulbar neuritis, retinitis, neuroretinitis, keratitis, central retinal vein occlusion, and exudative retinal detachment [8]. Cases of third nerve palsy, sixth nerve palsy, and facial nerve palsy have also been reported secondary to chikungunya [9,10].

There is no specific antiviral treatment for chikungunya. The mainstay of treatment is symptomatic relief, including hydration, rest, antipyretics, and non-steroidal anti-inflammatory drugs (NSAIDs). Ocular manifestations are treated with topical steroids, cycloplegic agents, and systemic steroids in cases of inflammation. Sixth nerve palsy typically resolves on its own within a few weeks to months. However, some patients may experience persistent symptoms or require further treatment [4].

Prevention is crucial in avoiding chikungunya. This can be achieved through vector control - eliminating breeding sites and using larvivorous fish in water tanks - mosquito repellent measures, and immunoprophylaxis with human anti-CHIKV immunoglobulins. Currently, there is no licensed vaccine available for chikungunya, but several are in development [11]. Public health campaigns and community participation are essential in controlling outbreaks.

It is uncertain whether transient blood pressure or metabolic changes, elevated intracranial pressure, or the virus itself directly led to ischemia. In this case, the patient had hypertension, which may have contributed to the abducens nerve palsy. High blood pressure can lead to microvascular ischemia, cerebrovascular accidents, and increased intracranial pressure, all of which are recognized causes of cranial nerve palsies. Regardless, both the palsy and the symptoms resolved spontaneously within a few weeks. Therefore, chikungunya infection should be included in the differential diagnosis of infectious sixth nerve palsies.

While this article provides valuable insights, it is important to acknowledge that a single case report limits the generalizability of the findings and the absence of long-term follow-up leaves the complete progression of the disease in this patient unclear.

## Conclusions

Chikungunya is a viral disease that has become a significant public health concern in recent years. The disease is spread by the bite of an infected mosquito, typically *A. aegypti *or *A. albopictus*, and causes severe joint pain, fever, and rashes. While primarily known for causing fever and joint pain, chikungunya can also be associated with ocular and neurological complications, including sixth nerve palsy. Recognizing these complications is important for providing comprehensive care, and more research is needed to understand the pathophysiology of Chikungunya-related cranial nerve palsies, as well as randomized trials assessing the use of steroids for these complications. Continued research, improved vector control strategies, and public health education are essential to control the spread of the disease and reduce its burden on affected populations.
